# Earlier clinical response predicts low rates of radiographic progression in biologic-naïve patients with active psoriatic arthritis receiving guselkumab treatment

**DOI:** 10.1007/s10067-023-06745-y

**Published:** 2023-10-03

**Authors:** Philip J. Mease, Alice B. Gottlieb, Alexis Ogdie, Iain B. McInnes, Soumya D. Chakravarty, Emmanouil Rampakakis, Alexa Kollmeier, Xie L. Xu, May Shawi, Frederic Lavie, Mitsumasa Kishimoto, Proton Rahman

**Affiliations:** 1 Swedish Medical Center/Providence St. Joseph Health and University of Washington, Seattle, WA USA; 2 Department of Dermatology, Icahn School of Medicine at Mount Sinai, New York, NY USA; 3 Division of Rheumatology, University of Pennsylvania School of Medicine, Philadelphia, PA USA; 4 College of Medical Veterinary and Life Sciences, University of Glasgow, Glasgow, UK; 5 Janssen Scientific Affairs, LLC, Horsham, PA USA; 6 Drexel University College of Medicine, Philadelphia, PA USA; 7 Department of Pediatrics, McGill University, Montreal, QC Canada; 8 JSS Medical Research, Montreal, Canada; 9 Immunology, Janssen Research & Development, LLC, San Diego, CA USA; 10 Immunology, Janssen Research & Development, LLC, Titusville, NJ USA; 11 Janssen Cilag Global Medial Affairs, Immunology Global Medical Affairs, Issy Les Moulineaux, France; 12 Department of Nephrology and Rheumatology, Kyorin University School of Medicine, Mitaka, Tokyo, Japan; 13 Discipline of Medicine, Division of Rheumatology, Memorial University of Newfoundland, St. John’s, NF Canada

**Keywords:** DAPSA, Guselkumab, Psoriatic arthritis, Radiographic progression, Structural joint damage, vdH-S score

## Abstract

**Objective:**

Assess relationship between earlier clinical improvement and radiographic progression (RP) over 2 years in guselkumab-treated patients with active psoriatic arthritis (PsA).

**Method:**

Post hoc analyses combined data from DISCOVER-2 biologic-naïve adults with active PsA randomized to either guselkumab 100 mg every 4 weeks (Q4W) or guselkumab at W0, W4, then Q8W. Correlations (Spearman’s coefficient) between baseline disease parameters and total PsA-modified van der Heijde-Sharp (vdH-S) score were examined. Repeated-measures mixed models, adjusted for known RP risk factors, assessed the relationship between Disease Activity Index in PsA (DAPSA) improvement, DAPSA improvement exceeding the median or the minimal clinically important difference (MCID), or DAPSA low disease activity (LDA) at W8 and RP rate, assessed by change from baseline in vdH-S score through W100.

**Results:**

Baseline age, PsA duration, CRP level, and swollen joint count, but not psoriasis duration/severity, weakly correlated with baseline vdH-S score. Elevated baseline CRP (parameter estimate [β] = 0.17–0.18, *p* < 0.03) and vdH-S score (β = 0.02, *p* < 0.0001) significantly associated with greater RP through W100. Greater improvement in DAPSA (β = -0.03, *p* = 0.0096), achievement of DAPSA improvement > median (least squares mean [LSM] difference: -0.66, *p* = 0.0405) or > MCID (-0.67, *p* = 0.0610), or DAPSA LDA (-1.44, *p* = 0.0151) by W8 with guselkumab significantly associated with less RP through W100. The effect of W8 DAPSA LDA on future RP was strengthened over time among achievers vs. non-achievers (LSM difference enhanced from -1.05 [*p* = 0.0267] at W52 to -1.84 [*p* = 0.0154] at W100).

**Conclusions:**

In guselkumab-treated patients with active PsA, earlier improvement in joint symptoms significantly associated with lower RP rates through 2 years, indicating blockade of the IL-23 pathway may modify long-term disease course and prevent further joint damage.

**Table:** 

Key Points • *Greater improvement in DAPSA at Week 8 of guselkumab treatment was significantly associated with less progression of structural joint damage at 2 years in patients with active psoriatic arthritis (PsA).* • *Early control of peripheral joint disease activity with blockade of the IL-23 pathway may modify long-term PsA trajectory and prevent further joint damage.*

**Supplementary Information:**

The online version contains supplementary material available at 10.1007/s10067-023-06745-y.

## Introduction

Psoriatic arthritis (PsA) is a complex inflammatory disease affecting peripheral and axial joints, entheses, and tendons, as well as skin and nails [1]. Structural joint damage occurs in up to half of patients with PsA within 2 years of developing clinical symptoms [2], and can be characterized by bone erosion and joint space narrowing (JSN), as well as new bone formation [3]. A higher degree of structural joint damage measured with conventional radiographs correlates with greater disability [4]; the Group for Research and Assessment of Psoriasis and Psoriatic Arthritis (GRAPPA) and the European League Against Rheumatism (EULAR) treatment recommendations support optimization of functional status and prevention of structural damage as goals of therapy for patients with PsA [5, 6].

Interleukin (IL)-23 is an upstream regulatory cytokine that modulates the development of IL-17-producing T cell populations, including, for example T helper (Th)-17 cells, and innate lymphoid cells, key effectors of inflammation in the skin and synovium, and consequently, of joint damage in PsA [7]. Guselkumab, a fully human monoclonal antibody that targets the IL-23p19 subunit [8], was approved to treat adults with active PsA based on findings from the phase 3 DISCOVER-1 (NCT03162796) and DISCOVER-2 (NCT03158285) trials [9–11]. Treatment with guselkumab 100 mg, either every 4 or 8 weeks (Q4W or Q8W), significantly improved signs and symptoms of PsA through Week 24 [9, 10] with sustained improvements through 1 year in DISCOVER-1 [12] and 2 years in DISCOVER-2 [13, 14]. The primary endpoint in DISCOVER-1 and -2 of ≥ 20% improvement from baseline in American College of Rheumatology (ACR20) criteria, which primarily assess joint disease activity (8), was achieved by significantly greater proportions of patients receiving guselkumab Q4W and Q8W compared with placebo at Week 24 [9, 10]. Proportions of guselkumab-randomized patients achieving ACR20/50/70 responses were maintained or increased through 1 year in DISCOVER-1 [12] and 2 years in DISCOVER-2 [13, 14]. The safety profile of guselkumab was similar to that of placebo through Week 24, and rates of adverse events, including those leading to discontinuation and serious infections, remained low, with no new safety concerns reported through 1 year (DISCOVER-1) [12] and 2 years (DISCOVER-2) [13, 14]. Robust responses on various composite PsA disease indices, including the Disease Activity Index in PsA (DAPSA), were observed as early as Week 8 in both guselkumab groups, with similar proportions of patients receiving guselkumab Q4W and Q8W achieving low levels of disease activity at 24 weeks [15]. In the majority of patients, response rates were maintained or increased at 1 year [15]. In the DISCOVER-2 trial of biologic-naïve patients with active PsA, guselkumab Q4W showed significantly less progression of structural damage at Week 24 compared with placebo [10], and low rates of radiographic progression (RP) were observed through 2 years of guselkumab treatment regardless of the dosing regimen [13, 14, 16]. Furthermore, patients receiving guselkumab Q4W or Q8W who achieved clinical responses across several global measures of PsA disease activity or normalized physical function at 1 or 2 years of treatment, showed less RP through Week 100 compared with nonresponders [16]. Supporting these findings, levels of inflammatory biomarkers, including C-reactive protein (CRP), and biomarkers of collagen degradation across diseases with musculoskeletal involvement, such as tissue metabolite of type I collagen (C1M), both known predictors of structural damage [17–21], were significantly suppressed at 24 weeks of guselkumab treatment with sustained reductions through 1 year [22, 23].

Given the association between persistent inflammation and joint damage in PsA [17–19, 22–25], the objective of this post hoc analysis was to determine whether earlier (by Week 8) clinical improvement in peripheral joint disease activity predicts RP through 2 years among biologic-naïve patients with active PsA treated with guselkumab.

## Methods

### Data source

This was a post hoc analysis of the phase 3 DISCOVER-2 trial. Details of the DISCOVER-2 study design, inclusion and exclusion criteria, a priori analyses, and primary efficacy and safety results have been reported [10, 13, 14]. Briefly, DISCOVER-2 was a double-blind, placebo-controlled, multi-center, phase 3 trial with biologic-naïve patients randomly allocated (1:1:1) to receive subcutaneous injections of guselkumab 100 mg Q4W, guselkumab 100 mg at Weeks 0 and 4 and Q8W thereafter, or placebo with crossover to guselkumab 100 mg Q4W at Week 24 [10, 13, 14]. Patients eligible for DISCOVER-2 were ≥ 18 years of age, with active PsA (≥ 5 swollen [0-66] and ≥ 5 tender [0-68] joint counts [SJC/TJC] and CRP level ≥ 6.0 mg/L) at screening, current or documented history of psoriasis, an inadequate response or intolerance to standard nonbiologic treatment (nonsteroidal anti-inflammatory drugs [NSAIDs], conventional synthetic disease-modifying antirheumatic drugs [csDMARDs], and apremilast), and no previous exposure to biologics or Janus kinase inhibitors (JAKi) [10]. Patients included in this post hoc analysis were those randomized to receive guselkumab 100 mg Q4W or Q8W.

### Study measures

Radiographs of the hands and feet obtained at Weeks 0, 24, 52 and 100 or at early discontinuation (if applicable) were scored in three separate reading sessions. The first reading session, comprising the primary radiographic endpoint analysis, included radiographs from Weeks 0 and 24 or at discontinuation if prior to Week 24; radiographs from Weeks 0, 24, and 52 or at discontinuation if between Weeks 24 and 52 were included in reading session 2; and radiographs from all time points including Week 100 were included in reading session 3 and are the subject of the current analyses. Radiographs were independently evaluated by two central readers with a third reader for adjudication [10, 13, 14]. Readers were blinded to treatment group and time points when scoring films using the van der Heijde-Sharp (vdH-S) score modified for PsA with the addition of distal interphalangeal joints of hands and pencil-in-cup/gross osteolysis deformities [26]. The PsA-modified vdH-S score is a composite score of the number and size of joint erosions (score 0–320) and the degree of JSN in the hands, wrists, and feet (score 0–208) with a maximum score of 528 [26]. Higher vdH-S scores indicate more radiographic damage [26]. RP from baseline through Week 100 was assessed by the change in the total PsA-modified vdH-S score.

With a focus on peripheral joint activity, clinical response at Week 8 was assessed with the DAPSA, a validated composite measure commonly used in clinical practice, rather than other composite measures that include additional disease manifestations [27]. DAPSA is calculated by summing individual scores for TJC (0–68), SJC (0–66), patient global assessment of arthritis using a visual analog scale (VAS; 0–10 cm), patient assessment of pain (VAS 0–10 cm), and CRP level (mg/dL) [28]. DAPSA disease activity states are defined as follows: remission (score ≤ 4), low disease activity (LDA, score > 4 and ≤ 14), moderate disease activity (ModDA, score > 14 and ≤ 28), and high disease activity (HDA, score > 28) [27]. The DAPSA minimal clinically important difference (MCID) has been previously defined as 7.25 [29].

### Data analysis

Data were combined for patients randomized to receive guselkumab 100 mg Q4W or Q8W, as similar clinical efficacy and rates of RP were observed through Week 100 in DISCOVER-2, regardless of guselkumab dosing regimen [10, 13, 14]. Observed changes in PsA-modified vdH-S scores from reading session 3, employing multiple imputation by fully conditional specification for missing radiographic data, were utilized in all analyses. Spearman’s rank-order correlation coefficient was used to examine the relationship between baseline disease parameters in the combined guselkumab Q4W and Q8W groups including PsA duration, CRP level, age, SJC (0-66), psoriasis duration, and Psoriasis Area and Severity Index (PASI) score and baseline total PsA-modified vdH-S score.

The associations between Week 8 change in DAPSA score as a continuous variable, DAPSA improvement greater than the median, DAPSA improvement more than the MCID (7.25), as well as achievement of DAPSA LDA (≤ 14), and the time-averaged changes in total PsA-modified vdH-S score from baseline to Weeks 52 and 100 were analyzed using multivariate mixed models for repeated measures accounting for known risk factors of RP including baseline age, sex, baseline PsA-modified vdH-S score, and baseline CRP level [18, 19, 24, 25, 30]. The interaction of clinical improvement at Week 8 with time was also tested and was included in the model if found to be statistically important (defined as *p* < 0.1). In addition to saturated models that included all covariates, reduced models were produced as confirmatory analyses, using stepwise backward variable selection with the Wald method and a p-value of 0.05 for removal of variables. In these analyses, the clinical improvement at Week 8 was forced into the model if it was not statistically significant. All analyses were performed using SAS software, version 9.4 (SAS Institute, Inc.; Cary, NC).

## Results

A total of 739 biologic-naïve patients with active PsA were randomized and treated in DISCOVER-2, of whom 245, 248, and 246 received guselkumab 100 mg Q4W, Q8W or placebo, respectively. Baseline patient demographics and disease characteristics were generally well balanced across treatment groups, and disease activity measures were consistent with active PsA (Table 1). Regarding known risk factors of radiographic outcomes, across treatment groups in the overall population, 53% of patients were male, the mean age was 45–46 years, and the median CRP level was 12.0–13.0 mg/L. Mean baseline vdH-S scores in reading session 3 were similar to those in reading session 1. Among the 664 patients (221, 228, and 215 patients in the guselkumab Q4W, Q8W, and placebo crossover groups, respectively) included in reading session 3 the mean PsA-modified vdH-S scores ranged from 23.9–28.0.

**Table 1:** Baseline demographics and disease characteristics

	Guselkumab 100 mg		Placebo
	Q4W	Q8W	
Randomized and treated patients, *N*	245	248	246
Age, years	45.9 (11.5)	44.9 (11.9)	46.3 (11.7)
Male, *n* (%)	142 (58)	129 (52)	117 (48)
Body weight, kg	85.8 (19.5)	83.0 (19.3)	84.0 (19.7)
PsA disease characteristics			
PsA disease duration, years	5.5 (5.9)	5.1 (5.5)	5.8 (5.6)
SJC (0–66)	12.9 (7.8)	11.7 (6.8)	12.3 (6.9)
TJC (0–68)	22.4 (13.5)	19.8 (11.9)	21.6 (13.1)
Psoriatic BSA, % (0–100)	18.2 (20.0)	17.0 (21.0)	17.1 (20.0)
IGA score of 3 or 4 (0–4), *n* (%)	117 (48)	108 (44)	115 (47)
CRP, mg/L, (normal ≤ 2.87 [19]), median (IQR)	12.0 (6.0–23.0)	13.0 (7.0–25.0)	12.0 (5.0–26.0)
DAPSA score^a^	49.7 (21.1)	46.3 (19.4)	48.8 (19.4)
Presence of enthesitis, (%)	69	64	72
LEI score (1–6)	3.0 (1.7)	2.6 (1.5)	2.8 (1.6)
Presence of dactylitis, (%)	49	45	40
DSS (1–60)	8.6 (9.6)	8.0 (9.6)	8.4 (9.3)
PsA-modified vdH-S score			
Reading session 1^b^, *N*	245	248	246
Total score (0–528)	27.2 (42.3)	23.0 (37.7)	23.8 (37.8)
Erosion score (0–320)	13.3 (22.4)	11.6 (20.3)	11.0 (19.1)
JSN score (0–208)	13.9 (21.5)	11.5 (18.3)	12.7 (19.9)
Reading session 3^c^, *N*	221	228	215
Total score (0–528)	28.0 (43.6)	23.9 (40.4)	25.6 (42.4)
Erosion score (0–320)	14.2 (23.3)	12.0 (21.9)	12.1 (21.9)
JSN score (0–208)	13.8 (21.8)	11.9 (19.5)	13.5 (21.6)
HAQ-DI score (0–3)	1.2 (0.6)	1.3 (0.6)	1.3 (0.6)
PASI score (0–72)	10.8 (11.7)	9.7 (11.7)	9.3 (9.8)

Table adapted from [10, 16]. Data are mean (SD) unless otherwise stated

^a^DAPSA score: remission ≤ 4, low disease  > 4 to ≤ 4, moderate disease > 14 to ≤ 28, high disease activity > 28

^b^Reading session 1 included randomized patients who received ≥ 1 administration of study drug (partial or complete) and had radiographic images obtained at Weeks 0 and 24 (or at discontinuation prior to Week 24)

^c^Reading session 3 included patients continuing study treatment at Week 52 with images at Weeks 0, 24, 52 and 100 (or at discontinuation after Week 52)

BSA = body surface area; CRP = C-reactive protein; DAPSA = Disease Activity Index for PsA; DSS = Dactylitis Severity Score; HAQ-DI = Health Assessment Questionnaire-Disability Index; IGA = Investigator’s Global Assessment; IQR = interquartile range; JSN = joint space narrowing; LEI = Leeds Enthesitis Index; PASI = Psoriasis Area and Severity Index; PsA = psoriatic arthritis; Q4W = every 4 weeks; Q8W = every 8 weeks; SJC = swollen joint count; TJC = tender joint count; vdH-S = van der Heijde-Sharp

Robust patient retention was observed in DISCOVER-2 with 94% and 90% of guselkumab-randomized patients completing study treatment through 1 and 2 years, respectively [13, 14].

### Correlation of baseline PsA-modified vdH-S scores with baseline PsA parameters

Among all patients in the combined guselkumab group, PsA and PsO duration, CRP level, age, and SJC at baseline were weakly correlated with baseline PsA-modified vdH-S total score. No correlation was observed between baseline PASI and vdH-S scores (Table 2).

**Table 2:** Correlation between baseline total PsA-modified vdH-S score and known baseline risk factors of radiographic progression among patients randomized to receive guselkumab Q4W or Q8W (*N* = 449)

Parameter at Baseline	Spearman's Rho	*p*-value
PsA duration	0.37	< 0.0001
CRP level	0.28	< 0.0001
Age	0.27	< 0.0001
SJC (0–66)	0.26	< 0.0001
PsO duration	0.21	< 0.0001
PASI score	0.03	0.5153

CRP = C-reactive protein; PASI = Psoriasis Area and Severity Index; PsA = psoriatic arthritis; PsO = psoriasis; Q4W = every 4 weeks; Q8W = every 8 weeks; SJC = swollen joint count; vdH-S = van der Heijde-Sharp

### Impact of known baseline risk factors of RP on total PsA-modified vdH-S scores through Week 100

In all models, elevated baseline CRP concentration and higher baseline PsA-modified vdH-S score were significantly associated with greater RP through Week 100 (CRP β range: 0.17–0.18, vdH-S score β = 0.02) (Table 3, Supplementary Tables S1-S3). Older age at baseline was consistently, although not significantly, associated with less RP (β = -0.02), while a pattern of greater progression of structural damage was associated with male sex (β range: 0.52–0.56) (Table 3, Supplementary Tables S1-S3).

**Table 3:** Multivariate association of DAPSA improvement at Week 8 and known baseline risk factors of radiographic progression with change in total PsA-modified vdH-S score through Week 100 among patients randomized to receive guselkumab Q4W or Q8W (*N* = 440)

	Saturated model		Reduced model	
Covariates	β	*p*-value	β	*p*-value
DAPSA improvement at Week 8	-0.03	0.0096	-0.03	0.0119
Baseline age	-0.02	0.2008	–	–
Baseline vdH-S score	0.02	< 0.0001	0.02	< 0.0001
Baseline CRP level	0.18	0.0121	0.22	0.0022
Baseline DAPSA score	0.01	0.5661	–	–
Male sex	0.56	0.0793	–	–

CRP = C-reactive protein; DAPSA = Disease Activity Index for PsA; PsA = psoriatic arthritis; vdH-S = van der Heijde-Sharp

### Associations of DAPSA improvement at Week 8 with change in the total PsA-modified vdH-S scores through Week 100

At Week 8, after receiving guselkumab at Week 0 and Week 4, 211 (42.8%) patients (combined guselkumab group) achieved median DAPSA improvement (decrease ≥ 12.55), 351 (71.2%) achieved DAPSA MCID (decrease ≥ 7.25), and 86 (17.4%) achieved DAPSA LDA.

**Fig. 1:**
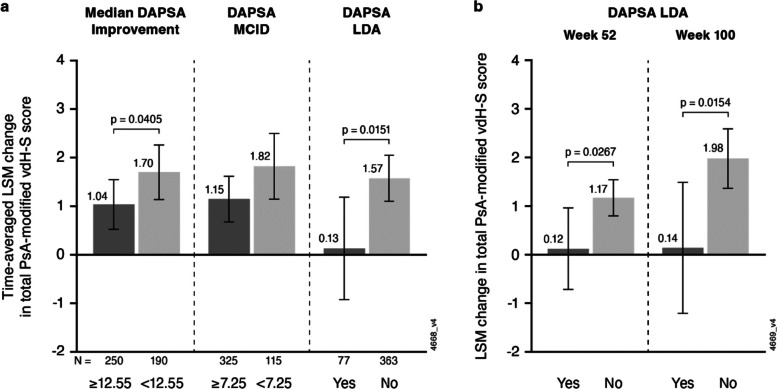
Multivariate association of DAPSA endpoints at Week 8 with change in total PsA-modified vdH-S score through Week 100 in guselkumab-randomized patients (saturated models). **a**) Impact of achieving DAPSA endpoints at Week 8 on time-averaged LSM changes from baseline to Weeks 52 and 100. **b**) Impact of achieving DAPSA LDA at Week 8 on LSM changes from baseline to Weeks 52 and 100. LSM changes in total PsA-modified vdH-S score were derived from mixed models adjusted for achievement of DAPSA endpoints at Week 8 and baseline age, sex, baseline CRP level, baseline vdH-S score, and baseline DAPSA scores**.** For DAPSA LDA**,** the interaction of endpoint achievement with time was also included in the model. CRP = C-reactive protein; DAPSA = Disease Activity Index for PsA; LDA = low disease activity; LSM = least squares mean; MCID = minimal clinically important difference; PsA = psoriatic arthritis; vdH-S = van der Heijde-Sharp

Saturated models demonstrated that among guselkumab-randomized patients with radiographic assessment at Week 100, greater improvement in DAPSA score at Week 8 was significantly associated with less RP of joint damage through Week 100 (parameter estimate [β] = -0.03, *p* = 0.0096) after adjusting for sex and baseline DAPSA, PsA-modified vdH-S score, age, and CRP level (Table 3). Achieving a DAPSA improvement greater than the median at Week 8 significantly associated with less RP through Week 100 (least squares mean [LSM] difference in achievers vs. non-achievers: -0.66, *p* = 0.0405), with corresponding LSM changes in the total PsA-modified vdH-S score of 1.04 vs. 1.70 among those achieving the median DAPSA improvement vs. patients who did not, respectively (Fig. 1a); DAPSA improvement exceeding the MCID threshold at Week 8 numerically associated with less RP (LSM difference: -0.67, *p* = 0.0610) through Week 100 (Supplementary Table S2 & Fig. 1a). Achievement of DAPSA LDA at Week 8 was significantly associated with less RP through Week 100 (LSM difference: -1.44, *p* = 0.0151) (Supplementary Table S3 & Fig. 1a). Furthermore, a statistically important interaction between time and achievement of DAPSA LDA at Week 8 was noted, where the impact of DAPSA LDA at Week 8 on RP strengthened over time, with the LSM difference in achievers vs. non-achievers enhanced from Week 52 (-1.05, *p* = 0.0267) to Week 100 (-1.84, *p* = 0.0154) (Fig. 1b). The interaction of time with DAPSA improvement exceeding the median or MCID thresholds at Week 8 was not statistically important.

Findings of the reduced models employing backward selection of variables were consistent with those of the saturated models (Table 3, Supplementary Tables S1-S3, Supplementary Fig. S1).

## Discussion

This post hoc analysis of the phase 3 DISCOVER-2 trial data revealed that, in patients with active PsA treated with guselkumab, achievement of earlier (Week 8) meaningful improvement in DAPSA score was significantly associated with less RP through 2 years of treatment. Achievement of DAPSA score exceeding the Week 8 median and MCID improvement thresholds, as well as of attainment of DAPSA LDA at Week 8, was associated with a pattern of less RP through Week 100. Moreover, achievement of DAPSA LDA at Week 8 was associated with less RP at Week 52, with further suppression of joint damage progression at Week 100. These findings were observed after adjusting for previously documented predictors of RP including older age, male sex, increased CRP levels, and higher baseline levels of structural damage [17–19, 24, 30]. Considering no notable differences in RP were observed between the guselkumab Q4W and Q8W dosing regimens through 2 years and consistent early DAPSA response rates in both treatment groups, the associations identified in the combined analyses are anticipated to also apply to the individual guselkumab dosing regimens.

The DAPSA score is a composite measure of the musculoskeletal domain of PsA and is based in part on an evaluation of actively inflamed joints and CRP level, both shown to be predictors of radiographic damage [17–19, 24]. A previous study investigating the relationship between DAPSA activity states and joint damage progression in PsA showed that the degree of functional impairment and probability of RP increase with more active disease, and higher DAPSA scores at 6 months significantly correlated with RP through 1 year [31]. In alignment with these findings, the present study demonstrated that early improvement in joint symptoms as assessed with DAPSA following guselkumab therapy is associated with less RP through 2 years in biologic-naive patients with active PsA. These data suggest that active inflammation driven by proinflammatory cytokines, including IL-23, may lead to joint damage and support blockade of the IL-23 pathway with guselkumab as an effective means of modulating disease activity and improving long-term prognosis. Among patients in DISCOVER-2, serum concentrations of C1M, a type I collagen metabolite and a direct marker of inflammation-driven bone degradation, decreased significantly with guselkumab Q4W and Q8W compared with placebo at Week 24 and reductions were maintained at Week 52 [23, 32]. C1M has been shown to be predictive of structural damage in rheumatoid arthritis [20, 21] and may hold similar prognostic value in PsA. Similarly, concentrations of CRP, a marker of both acute and chronic inflammation used for diagnosis and monitoring of PsA [33], were significantly reduced as early as Week 4 with guselkumab in DISCOVER-1 and -2, achieving levels comparable to those of healthy controls at Week 24 of treatment [22]. Recent studies have, however, challenged the usefulness of CRP in assessing disease activity among PsA patients by comparing DAPSA with cDAPSA, which omits CRP [34–36]. A cross-sectional analysis of a real-world PsA population found that approximately half of patients with cDAPSA scores indicative of moderate-to-high disease activity had normal CRP levels [34]. Data from a real-world cohort and an observational clinical study showed a high correlation between DAPSA and cDAPSA scores, with patients achieving remission regardless of CRP levels [35, 36]. These findings suggest that inclusion of laboratory markers in disease target measures may not be necessary; moreover, a clinical assessment may be more feasible in routine practice. To further define the relationship between early improvement in disease activity and long-term structural joint damage, the association of cDAPSA with RP is a suggested focus of future investigation among patients with PsA treated with guselkumab.

A number of treatment options are available in PsA across therapeutic classes including anti-IL-12/23 agents, IL-17A inhibitors acting downstream of IL-23, tumor necrosis factor inhibitors (TNFi), small molecules, and cytotoxic T-lymphocyte-associated antigen 4 inhibitors, all with demonstrated ability to significantly improve skin and joint symptoms [1]. In a recent network meta-analysis comparing the effectiveness of guselkumab and 15 other targeted PsA therapies through the end of the placebo-controlled periods (12–24 weeks), guselkumab demonstrated joint efficacy comparable to most interventions, with changes in vdH-S scores similar to IL-17A, JAKi, and subcutaneous TNFi [37]. The present analysis further informs our understanding of the potential relationship between early modification of PsA joint disease activity to a milder trajectory with the selective IL-23 inhibitor guselkumab, and sustained attenuation of long-term structural joint damage.

Consistent with previous studies, the present analysis showed that elevated baseline CRP concentration, as well as higher baseline radiographic score, were predictive of future joint damage [17, 18, 24], further supporting a role for inflammation in structural damage progression [17, 19]. In the current analyses, RP was not significantly impacted by patient age, and while some studies have identified age as a predictor of such damage [24], others found that clinical features may have only a limited ability to discriminate patients at risk of joint damage progression in PsA [18, 38]. In agreement with previous observations of sex differences in PsA disease manifestations [30, 39], the current analyses from DISCOVER-2 identified a trend toward an association of male sex with more RP, emphasizing the importance of integrating sex differences into interpretation of therapeutic response [40].

Several limitations of the current post hoc analyses should be noted. Results of analyses conducted on a clinical trial population enriched for patients with a higher risk of RP and no history of biologic therapy may have limited external validity to the broader population of patients with PsA. A follow-up duration of 100 weeks may not be sufficient to evaluate RP in PsA; hence, the ongoing APEX phase 3b study (NCT04882098), designed to evaluate guselkumab efficacy in the inhibition of RP in patients with PsA through 3 years, may offer greater insight into long-term effects of guselkumab. These analyses were conducted post hoc, and the DISCOVER-2 study was not adequately powered to answer the question under study, which may have resulted in lack of statistical significance for certain associations. Strengths of the present analysis include the low guselkumab discontinuation rates in the DISCOVER-2 trial, and the use of DAPSA, a validated PsA assessment instrument. Although safety analyses were out of scope for these post hoc analyses, previous reports have demonstrated the favorable safety profile of guselkumab during the placebo-controlled study period, with consistent findings through up to 2 years of follow-up among patients with PsA [41, 42].

## Conclusions

In this post hoc analysis of data from the phase 3 DISCOVER-2 study of guselkumab-treated biologic-naïve patients with active PsA, earlier (Week 8) DAPSA improvement was a significant predictor of less RP through 2 years. Thus, the early and sustained control of joint disease activity with guselkumab may modify the overall long-term PsA disease trajectory to a milder course.

### Supplementary Information

Below is the link to the electronic supplementary material.

Supplementary file1 (DOCX 152 KB)

## Data Availability

The data sharing policy of Janssen Pharmaceutical Companies of Johnson & Johnson is available at https://www.janssen.com/clinical-trials/transparency. As noted on this site, requests for access to the study data can be submitted through the Yale Open Data Access (YODA) Project site at http://yoda.yale.edu.
