# Supplementary material for: Earlier clinical response predicts low rates of radiographic progression in biologic-naïve patients with active psoriatic arthritis receiving guselkumab treatment
Source: Clin Rheumatol. 2023 Oct 3;43(1):241–9. doi: 10.1007/s10067-023-06745-y (PMC10774160; doi:10.1007/s10067-023-06745-y)
Supplement: Supplementary file 1 — Supplementary file1 (DOCX 152 KB) [file 10067_2023_6745_MOESM1_ESM.docx]

# Supplementary Materials

Supplementary Table S1 Multivariate association of median DAPSA improvement at Week 8 and known baseline risk factors of radiographic progression with change in total PsA-modified vdH-S score over 100 weeks among guselkumab-randomized patients (N=440)

|  | Saturated model | | Reduced model | |  |
| --- | --- | --- | --- | --- | --- |
| Covariates | β | p-value | β | p-value | |
| Median DAPSA improvement at Week 8 | -0.66 | 0.0405 | -0.62 | 0.0510 | |
| Baseline age | -0.02 | 0.1401 | -- | -- | |
| Baseline vdH-S score | 0.02 | <0.0001 | 0.02 | <0.0001 | |
| Baseline CRP level | 0.18 | 0.0157 | 0.21 | 0.0039 | |
| Baseline DAPSA score | 0 | 0.9832 | -- | -- | |
| Male sex | 0.53 | 0.0966 | -- | -- | |

CRP=C-reactive protein; DAPSA=Disease Activity Index for PsA; PsA=psoriatic arthritis; vdH-S=van der Heijde-Sharp

Supplementary Table S2 Multivariate association of DAPSA MCID at Week 8 and known baseline risk factors of radiographic progression with change in total PsA-modified vdH-S score through Week 100 among guselkumab-randomized patients (N=440)

|  | Saturated model | | Reduced model | |
| --- | --- | --- | --- | --- |
| Covariates | β | p-value | β | p-value |
| DAPSA MCID at Week 8 | -0.67 | 0.0610 | -0.66 | 0.0647 |
| Baseline age | -0.02 | 0.1706 | -- | -- |
| Baseline vdH-S score | 0.02 | <0.0001 | 0.02 | <0.0001 |
| Baseline CRP level | 0.18 | 0.0155 | 0.20 | 0.0042 |
| Baseline DAPSA score | 0 | 0.8717 | -- | -- |
| Male sex | 0.52 | 0.0994 | -- | -- |

CRP=C-reactive protein; DAPSA=Disease Activity Index for PsA; MCID=minimal clinically important difference; PsA=psoriatic arthritis; vdH-S=van der Heijde-Sharp

Supplementary Table S3 Multivariate association of DAPSA LDA at Week 8 and known baseline risk factors of radiographic progression with change in total PsA-modified vdH-S score through Week 100 among guselkumab-randomized patients (N=440)

|  | Saturated model | | Reduced model | |
| --- | --- | --- | --- | --- |
| Covariates | β | p-value | β | p-value |
| DAPSA LDA at Week 8 | -1.84 | 0.0154 | -1.73 | 0.0208 |
| DAPSA LDA at Week 8 * time | 0.79 | 0.0694 | 0.79 | 0.0688 |
| Baseline age | -0.02 | 0.2392 | -- | -- |
| Baseline vdH-S score | 0.02 | <0.0001 | 0.02 | <0.0001 |
| Baseline CRP level | 0.17 | 0.0241 | 0.18 | 0.0107 |
| Baseline DAPSA score | -0.01 | 0.3906 | -- | -- |
| Male sex | 0.54 | 0.0899 | -- | -- |

CRP=C-reactive protein; DAPSA=Disease Activity Index for PsA; LDA=low disease activity; PsA=psoriatic arthritis; vdH-S=van der Heijde-Sharp

Supplementary Fig. S1 Multivariate association of DAPSA endpoints at Week 8 with change in total PsA-modified vdH-S score through Week 100 in guselkumab-randomized patients (reduced models). a) Impact of achieving DAPSA endpoints at Week 8 on time-averaged LSM changes from baseline to Weeks 52 and 100. b) Impact of achieving DAPSA LDA at Week 8 on LSM changes from baseline to Weeks 52 and 100.

LSM changes in total PsA-modified vdH-S score were derived from mixed models adjusted for achievement of DAPSA endpoints at Week 8 and variables selected following stepwise backward selection among the following: age, sex, baseline CRP level, baseline vdH-S score, and baseline DAPSA scores. For DAPSA LDA, the interaction of endpoint achievement with time was also included in the model. CRP=C-reactive protein; DAPSA=Disease Activity Index for PsA; LDA=low disease activity; LSM=least squares mean; MCID=minimal clinically important difference; PsA=psoriatic arthritis; vdH-S=van der Heijde-Sharp

**
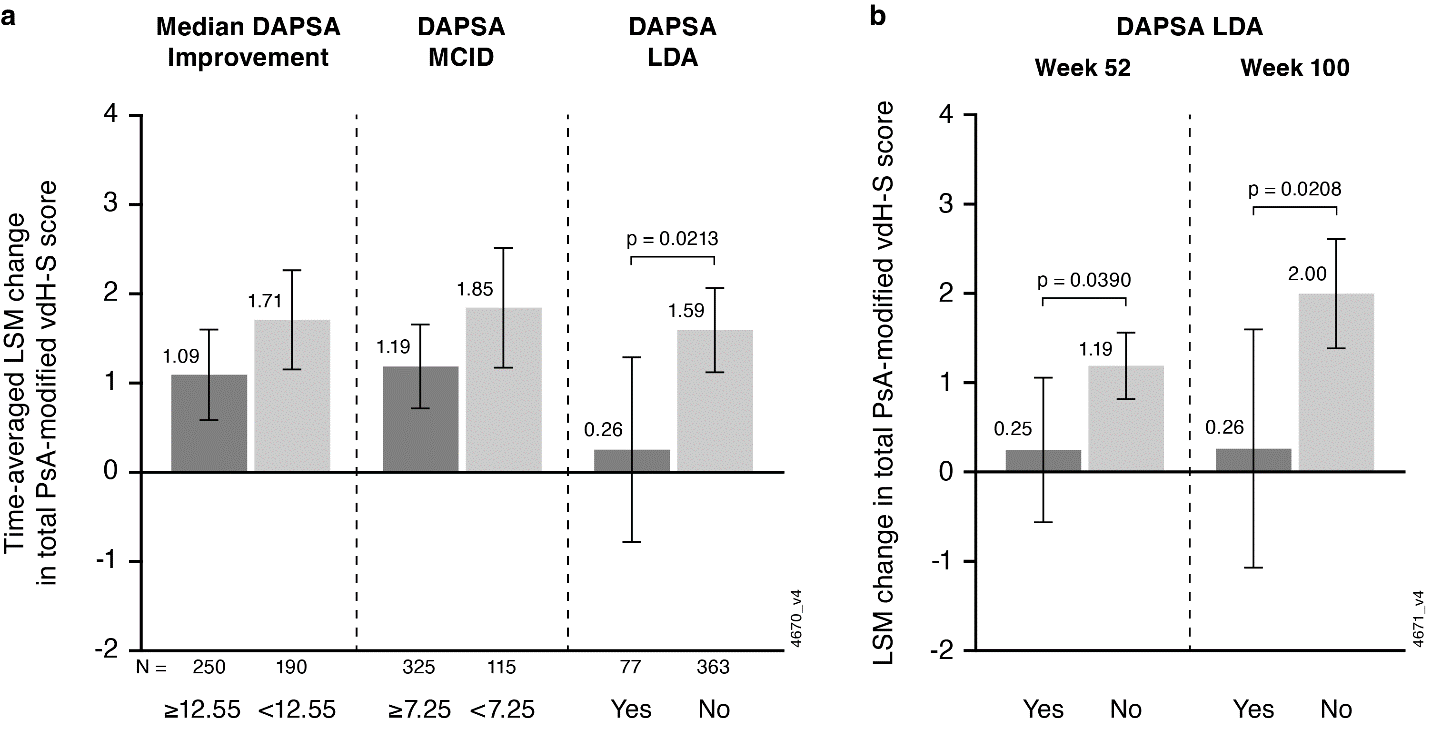
**
